# Treatment success in pragmatic randomised controlled trials: a review of trials funded by the UK Health Technology Assessment programme

**DOI:** 10.1186/1745-6215-12-S1-A97

**Published:** 2011-12-13

**Authors:** Louise Dent, James Raftery

**Affiliations:** 1 University of Southampton Clinical Trials Unit, MP131, Southampton General Hospital, SO16 6YD, UK; 2 NIHR Evaluation, Trials and Studies Coordinating Centre, University of Southampton, Southampton, SO16 7NS, UK

## Background

Equipoise implies that given a random unbiased sample of trials, no significant difference would be expected in the proportion favouring the new treatment to the proportion favouring the standard treatment [1-3]. Previous research reviewed treatment success and whether the collective uncertainty principle is met in RCTs in the US National Cancer Institute portfolio [4-6]. This paper classifies clinical trials funded by the UK HTA programme by results using the method applied to the US Cancer Institute trials, and compares the two portfolios [7].

## Methods

Data on all completed randomised controlled trials funded by the HTA programme 1993-2008 were extracted. Each trial's primary results was classified into six categories; 1) statistically significant in favour of the new treatment, 2) statistically significant in favour of the control treatment 3) true negative, 4) truly inconclusive, 5) inconclusive in favour of new treatment or 6) inconclusive in favour of control treatment. Trials were classified by comparing the 95% confidence interval for the difference in primary outcome to the difference specified in the sample size calculation. The results were compared with Djulbegovic's analysis of NCI trials.

## Results

Data from 51 superiority trials were included, involving over 48,000 participants and a range of diseases and interventions. 85 primary comparisons were available because some trials had more than two randomised arms or had several primary outcomes. The new treatment had superior results (whether significant or not) in 61% of the comparisons (52/85 95% CI 49.9% to 71.6%). The results were conclusive in 46% of the comparisons (19% statistically significant in favour of the new treatment, 5% statistically significant in favour of the control and 22% true negative). The results were classified as truly inconclusive (i.e. failed to answer the question asked) for 24% of comparisons (20/85). HTA trials included fewer truly inconclusive and statistically significant results and more results rated as true negative than NCI trials.

## Conclusions

The pattern of results in HTA trials is similar to that of the National Cancer Institute portfolio. Differences that existed were plausible given the differences in the types of trials -HTA trials are more pragmatic. The results indicate HTA trials are compatible with equipoise. This classification usefully summarises the results from clinical trials and enables comparisons of different portfolios of trials.

## References

[B1] Kumar A Soares H Wells R Clarke M Hozo L Bleyer A Are experimental treatments for cancer in children superior to established treatments? Observational study of randomised controlled trials by the Children's Oncology Group British Medical Journal 2005 331 7528 1295 1298B 10.1136/bmj.38628.561123.7C 16299015 PMC1298846

[B2] Djulbegovic B Articulating and responding to uncertainties in clinical research Journal of Medicine and Philosophy 2007 32 2 79 98 10.1080/03605310701255719 17454416

[B3] Djulbegovic B The paradox of equipoise: the principle that drives and limits therapeutic discoveries in clinical research Cancer Control 2009 16 4 342 347 19910921 10.1177/107327480901600409 PMC2782889

[B4] Djulbegovic B Kumar A Soares HP Hozo I Bepler G Clarke M Treatment success in cancer Archives of Internal Medicine 2008 168 6 632 42 10.1001/archinte.168.6.632 18362256 PMC2773511

[B5] Joffe S Harrington DP George SL Emanuel EJ Budzinski LA Weeks JC Satisfaction of the uncertainty principle in cancer clinical trials: retrospective cohort analysis BMJ 2004 328 7454 1463 10.1136/bmj.38118.685289.55 15163611 PMC428513

[B6] Soares HP Kumar A Daniels S Swann S Cantor A Hozo I Evaluation of new treatments in radiation oncology: are they better than standard treatments? JAMA 2005 293 8 970 978 10.1001/jama.293.8.970 15728168 PMC1779758

[B7] Dent L Raftery J Treatment success in pragmatic randomised controlled trials: a review of trials funded by the UK Health Technology Assessment programme Trials 2011 12 109 10.1186/1745-6215-12-109 21542934 PMC3113983

